# A Novel Expectation-Maximization-Based Blind Receiver for Low-Complexity Uplink STLC-NOMA Systems

**DOI:** 10.3390/s22208054

**Published:** 2022-10-21

**Authors:** Ki-Hun Lee, Bang Chul Jung

**Affiliations:** Department of Electronics Engineering, Chungnam National University, Daejeon 34134, Korea

**Keywords:** amplitude-shift keying (ASK), blind decoder, clustering, expectation-maximization (EM), Gaussian mixture model (GMM), Internet-of-things (IoT), low-complexity transceiver, space-time line code (STLC), uplink non-orthogonal multiple access (NOMA)

## Abstract

In this paper, we revisit a two-user space-time line coded uplink non-orthogonal multiple access (STLC-NOMA) system for Internet-of-things (IoT) networks and propose a novel low-complexity STLC-NOMA system. The basic idea is that both IoT devices (stations: STAs) employ amplitude-shift keying (ASK) modulators and align their modulated symbols to in-phase and quadrature axes, respectively, before the STLC encoding. The phase distortion caused by wireless channels becomes compensated at the receiver side with the STLC, and thus each STA’s signals are still aligned on their axes at the access point (AP) in the proposed uplink STLC-NOMA system. Then, the AP can decode the signals transmitted from STAs via a single-user maximum-likelihood (ML) detector with low-complexity, while the conventional uplink STLC-NOMA system exploits a multi-user joint ML detector with relatively high-complexity. We mathematically analyze the exact BER performance of the proposed uplink STLC-NOMA system. Furthermore, we propose a novel expectation-maximization (EM)-based blind energy estimation (BEE) algorithm to jointly estimate both transmit power and effective channel gain of each STA without the help of pilot signals at the AP. Somewhat interestingly, the proposed BEE algorithm works well even in short-packet transmission scenarios. It is worth noting that the proposed uplink STLC-NOMA architecture outperforms the conventional STLC-NOMA technique in terms of bit-error-rate (BER), especially with high-order modulation schemes, even though it requires lower computation complexity than the conventional technique at the receiver.

## 1. Introduction

The fifth-generation (5G) and beyond mobile communication systems with Internet-of-things (IoT) are vital infrastructures to realize seamless and hyper-connectivity networks. Future IoT applications will consist of a massive number of wireless devices; hence the next-generation networks, especially 6G, require much higher spectral- and energy-efficiency, connection density, and lower latency performance than 5G networks [1,2,3]. During a decade, non-orthogonal multiple access (NOMA) technologies have been vigorously studied to fulfill the requirements above and overcome the spectrum scarcity problem [4,5,6,7]. The basic concept of NOMA is to allow multiple users to share the same radio resources through power- or code-domain multiplexing. It has been found that NOMA can significantly improve spectral efficiency and network capacity in wireless communication systems [8,9]. More recently, various NOMA techniques have been investigated, applying grant-free multiple access [3,10], backscatter communication [11], reconfigurable intelligent surface (RIS) [12,13], relay-assisted network [14], unmanned aerial vehicle (UAV)-enabled network [15], visible light communication (VLC) [16], etc.

Space-time line code (STLC) is a symmetric communication technique of the well-known space-time block code (STBC) [17]. The STLC can achieve full-rate and full-diversity gain performance in multiple-input multiple-output (MIMO) systems, even though full channel state information (CSI) is available only at the transmitter (CSIT). As summarized in Refs. [18,19,20], its applications to various MIMO communication systems have been actively investigated. In particular, we have proposed an STLC-applied NOMA (STLC-NOMA) system for uplink IoT networks while raising some issues about the conventional uplink NOMA systems [21,22,23]. Most existing literature on the uplink NOMA assumes that CSI for all users is available at the base station (BS). However, it is practically infeasible for an access point (AP) to acquire the full CSI of a massive number of IoT devices (stations: STAs) due to the high signaling overhead for exchanging pilot signals [23]. We exploited the channel reciprocity property in the time-division duplex (TDD) system and applied STLC to NOMA-based uplink IoT networks. In conclusion, we have observed that each STA in the two-user uplink STLC-NOMA network can achieve the same bit-error-rate (BER) performance as the STLC using orthogonal resources as the signal-to-noise ratio (SNR) increases. Moreover, we have concluded that the uplink STLC-NOMA system is remarkably adequate for IoT networks in which two STAs share common radio resource blocks, considering the complexity and spatial diversity gain.

In this paper, we revisit the conventional two-user uplink STLC-NOMA system [23] and propose a novel low-complexity uplink STLC-NOMA system to strike the following shortcomings.

- Although the joint maximum-likelihood (ML) detector achieves optimal BER performance for uplink NOMA systems, it results in significant computational complexity;
- The complex constellation diagram at the AP makes it intractable for the AP to estimate the effective channel gain of each STA blindly.

To this end, instead of phase-shift keying (PSK) or quadrature amplitude modulation (QAM) with constellation rotation as in the conventional uplink STLC-NOMA system, we employ *M*-ary amplitude-shift keying (ASK) modulations for both STAs and align each STA’s modulated symbols on each axis in the in-phase and quadrature (I-Q) plane. Thanks to the fundamental nature of STLC, the phase distortion of the transmitted signals caused by wireless channels is compensated at the receiver. In other words, on the AP side, the signals of each STA are still aligned on their axis with only the channel gain applied; hence the AP can detect the transmitted signals of each STA through an ML detector without joint ML detection.

On the other hand, the AP requires each STA’s transmit power and effective channel gain called partial CSI to detect transmitted signals. This is indispensable in the uplink STLC-NOMA system for blindly decoding the signals transmitted from STAs, but it has not been well-elaborated in literature [22,23]. Therefore, we further design a blind energy estimation (BEE) scheme for the proposed low-complexity STLC-NOMA system based on expectation-maximization (EM) for Gaussian mixture model (GMM) [24,25]. Although a machine learning-based blind decoding method for STLC systems has been proposed in Ref. [26], our algorithm can be implemented without a learning engine; hence it has low-complexity and high cost-efficiency. More specifically, the AP exploits the EM algorithm that infers the parameters of a GMM as the blind transmit power and effective channel gain estimation method for both STAs. As a result, our main contributions in this paper can be summarized as follows.

- We proposed a novel low-complexity STLC-NOMA system for two-user uplink IoT networks. We employ amplitude-shift keying (ASK) modulators for both STAs, and align them respectively to each axis in the I-Q plane;
- We theoretically analyze the exact BER performance of the novel uplink STLC-NOMA system. The Monte-Carlo simulations validate that the mathematical BER expressions of the proposed system are precisely the same as the numerical results;
- We design an EM-based BEE method estimating the transmission power and effective channel gain of each STA at the AP. Then, the AP can detect the signals transmitted from the two STAs via an ML detector.

The remainder of this paper is organized as follows. In Section 2, we describe the system model of the proposed novel low-complexity uplink STLC-NOMA system. In Section 3, we mathematically analyze the exact BER performance and spatial diversity order for each STA. In Section 4, we design a blind energy estimation (BEE) scheme based on the EM for GMM. In Section 5, numerical results are presented. The conclusions are drawn in Section 6.

## 2. System Model

We consider an uplink STLC-NOMA network consisting of two single-antenna IoT devices (stations: STAs) and a dual-antenna access point (AP), where each STA has its local CSI and the AP does not have any CSI (CSIT) as in Ref. [23]. Although we consider a simple system model for the convenience of explanation, the framework proposed in this paper can be easily extended to the MIMO system model by employing the STLC encoder for multiple transmit antennas and low-rate STLC combiner for multiple receive antennas [17]. Unlike Ref. [23], both STAs employ *M*-ary amplitude-shift keying (ASK) modulators on the in-phase and quadrature axes, respectively. Without loss of generality, we assume that the first STA occupies the in-phase axis; thus, the second STA uses the quadrature axis. One frame of the STA consists of *L* symbols, where *L* is defined as the frame length, and block fading channels are assumed while at least one frame is transmitted, i.e., the wireless channels remain constant during a frame time. It is also assumed that two STAs in the network are synchronized by observing the common pilot signals periodically broadcast from the AP.

Both STAs simultaneously transmit their frame to the AP exploiting STLC over the same radio resource blocks. The STLC encoded signal for the l(∈{1,2,⋯,L})th symbol of the n(∈{1,2})th STA, denoted by $s_{n}^{\left(l\right)}$, can be expressed equivalently as follows:

(1) $$\begin{array}{cc} s_{n}^{\left(2l^{\prime }-1\right)} & =\sqrt{P_{n}}\frac{h_{n,1}^{*}\left(j^{n-1}x_{n}^{\left(2l^{\prime }-1\right)}\right)+h_{n,2}^{*}{\left(j^{n-1}x_{n}^{\left(2l^{\prime }\right)}\right)}^{*}}{\sqrt{|h_{n,1}{|}^{2}+{\left|h_{n,2}\right|}^{2}}},\\ s_{n}^{\left(2l^{\prime }\right)} & =\sqrt{P_{n}}\frac{h_{n,2}^{*}{\left(j^{n-1}x_{n}^{\left(2l^{\prime }-1\right)}\right)}^{*}-h_{n,1}^{*}\left(j^{n-1}x_{n}^{\left(2l^{\prime }\right)}\right)}{\sqrt{|h_{n,1}{|}^{2}+{\left|h_{n,2}\right|}^{2}}}, \end{array}$$

where $j\;(≜\sqrt{-1})$ represents the imaginary unit and $l^{\prime }\;\left(\in \left\{1,2,\cdots ,L/2\right\}\right)$ is defined to indicate the odd ($2l^{\prime }-1$) and even ($2l^{\prime }$) *M*-ASK symbols in the frame; $x_{n}^{\left(l\right)}$ denotes the *l*th normalized *M*-ASK symbol of the *n*th STA, such that $E[x_{n}^{\left(l\right)}]=1$, and $h_{n,m}$ denotes the wireless channel between the *n*th STA and the m(∈{1,2})th antenna of the AP. All channels are assumed to follow an independent and identically distributed (i.i.d.) complex Gaussian distribution with zero mean and unit variance, i.e., $h_{n,m}∼𝒞𝒩\left(0,1\right),\;\forall n,m$. As aforementioned, static channels are assumed for one frame, so $h_{n,m}$ remains constant for each frame of length *L* symbols. Furthermore, $P_{n}$ is the transmission power of the *n*th STA, such that $E[∥{\left[s_{n}^{\left(2l^{\prime }-1\right)},s_{n}^{\left(2l^{\prime }\right)}\right]}^{T}{∥}^{2}/2]=P_{n}$. Equation (1) shows that two consecutive *M*-ASK symbols are encoded into two STLC signals within the frame.

The received frame at the *m*th antenna of the AP, denoted by $y_{m}\;\left(={\left[y_{m}^{\left(1\right)},\cdots ,y_{m}^{\left(L\right)}\right]}^{T}\in C^{L\times 1}\right)$, is then written as follows:

(2) $$y_{m}=\sum_{n=1}^{2}\sqrt{\beta_{n}}\cdot h_{n,m}\cdot s_{n}+w_{m},$$

where $\beta_{n}$ is the large-scale fading component, $s_{n}\;\left(={\left[s_{n}^{\left(1\right)},\cdots ,s_{n}^{\left(L\right)}\right]}^{T}\in C^{L\times 1}\right)$ represents the transmit frame of the *n*th STA, and $w_{m}\;\left(=\left[w_{m}^{\left(1\right)},\cdots ,w_{m}^{\left(L\right)}\right]\in C^{L\times 1}\right)$ denotes the additive white Gaussian noise (AWGN) at the *m*th antenna of the AP that follows an i.i.d. $\mathrm{CN}(0,N_{0}\cdot I_{L})$, where $N_{0}$ and $I_{L}$ represent the noise power and the L×L identity matrix, respectively.

The AP combines the received signals in (2) according to the linear combining procedure of the STLC as follows:

(3) $$\begin{array}{cc} {\tilde{y}}_{2l^{\prime }-1} & =y_{1}^{\left(2l^{\prime }-1\right)}+{\left(y_{2}^{\left(2l^{\prime }\right)}\right)}^{*}=\sum_{n=1}^{2}\sqrt{P_{n}}\sqrt{\beta_{n}}∥h_{n}∥j^{n-1}x_{n}^{\left(2l^{\prime }-1\right)}+w_{1}^{\left(2l^{\prime }-1\right)}+{\left(w_{2}^{\left(2l^{\prime }\right)}\right)}^{*},\\ {\tilde{y}}_{2l^{\prime }} & ={\left(y_{2}^{\left(2l^{\prime }-1\right)}\right)}^{*}-y_{1}^{\left(2l^{\prime }\right)}=\sum_{n=1}^{2}\sqrt{P_{n}}\sqrt{\beta_{n}}∥h_{n}∥j^{n-1}x_{n}^{\left(2l^{\prime }\right)}+{\left(w_{2}^{\left(2l^{\prime }-1\right)}\right)}^{*}-w_{1}^{\left(2l^{\prime }\right)}, \end{array}$$

where ${\tilde{y}}_{l}$ denotes the *l*th STLC decoding signal and $h_{n}≜{\left[h_{n,1},h_{n,2}\right]}^{T}$. We can observe that the phase distortion of the transmitted signals caused by the wireless channels is completely compensated thanks to the fundamental nature of STLC, hence each STA’s symbols $x_{n}^{\left(l\right)}$ are still aligned for each axis in the I-Q plane. From (3), the STLC decoding frame at the AP, denoted by $\tilde{y}\;\left(={\left[{\tilde{y}}_{1},\cdots ,{\tilde{y}}_{l},\cdots ,{\tilde{y}}_{L}\right]}^{T}\in C^{L\times 1}\right)$, can be stated as

(4) $$\tilde{y}=\sqrt{P_{1}}\sqrt{\beta_{1}}∥h_{1}∥x_{1}+j\sqrt{P_{2}}\sqrt{\beta_{2}}∥h_{2}∥x_{2}+\tilde{w},$$

where $x_{n}\;\left(={\left[x_{n}^{\left(1\right)},\cdots ,x_{n}^{\left(L\right)}\right]}^{T}\in C^{L\times 1}\right)$ denotes the length-*L* frame transmitted from the *n*th STA, each consisting of *L* *M*-ASK symbols; and $\tilde{w}$ represents the equivalent AWGN vector at the AP following an i.i.d. $\mathrm{CN}(0,2N_{0}\cdot I_{L})$. Note that the noise variance is doubled due to the nature of STLC.

Let $\gamma_{n}$ be the effective channel gain of the *n*th STA defined as $\gamma_{n}≜{∥g_{n}∥}^{2}$, where $g_{n}\;\left(≜\sqrt{\beta_{n}}{\left[h_{n,1},h_{n,2}\right]}^{T}\in C^{2\times 1}\right)$ is the wireless channel vector including the large-scale fading component between the *n*th STA and the AP, i.e., $g_{n}∼\mathrm{CN}\left(0,\beta_{n}\cdot I_{2}\right)$. We will design a blind energy estimation (BEE) algorithm for the proposed uplink STLC-NOMA system in Section 4. This allows the AP to blindly estimate the transmit power and effective channel gain of each STA. The AP then detects the transmitted *M*-ASK symbols for all *n* and *l* through a simple maximum-likelihood (ML) detector from (4) as follows:

(5) $$\begin{array}{cc} {\hat{x}}_{1}^{\left(l\right)} & =\arg\min_{x\in X}{\left|ℜ\left[{\tilde{y}}_{l}\right]-\sqrt{P_{1}\gamma_{1}}x\right|}^{2},\;\mathrm{for}\;\mathrm{the}\;\mathrm{first}\;\mathrm{STA},\\ {\hat{x}}_{2}^{\left(l\right)} & =\arg\min_{x\in X}{\left|ℑ\left[{\tilde{y}}_{l}\right]-\sqrt{P_{2}\gamma_{2}}x\right|}^{2},\;\mathrm{for}\;\mathrm{the}\;\mathrm{second}\;\mathrm{STA}, \end{array}$$

where ℜ[·] and ℑ[·] denote the real and imaginary components of a complex number, respectively; ${\hat{x}}_{n}^{\left(l\right)}$ is the estimate of the *l*th symbol of the *n*th STA, and X is a set of the normalized *M*-ASK symbols defined as

$$X=\left\{x|x=\frac{2m-1-M}{\sqrt{\sum_{m=1}^{M}{\left(2m-1-M\right)}^{2}/M}},m=1,2,\cdots ,M\right\}.$$

It is worth noting that the proposed novel low-complexity STLC-NOMA system has O(L·M) computational complexity for the detector, whereas the conventional STLC-NOMA employing a joint ML detector has $O(L\cdot M^{2})$ complexity in the two-user uplink IoT network [23].

## 3. Performance Analysis

We mathematically analyze the performance of the proposed novel low-complexity uplink STLC-NOMA system employing *M*-ASK modulation. In particular, we derive the bit-error-rate (BER) performance and spatial diversity order of each STA. Since the signals of both STAs are aligned orthogonally to each other on the I-Q plane, the BER performance of each STA can be derived straightforwardly from the BER expression for the *M*-ary pulse amplitude modulation (PAM). Specifically, the BER of the *n*th STA, denoted by $P_{b,n}$, can be derived from Ref. [27] as follows:

(6) $$\begin{array}{cc} P_{b,n} & =E_{\gamma_{n}}\;\left[\frac{1}{M\log_{2}M}\sum_{k=1}^{\log_{2}M}\;\left[\;\sum_{i=0}^{(1-2^{-k})M-1}\;\left\{\;\Phi \left(i,k,m\right)\;\cdot \;\mathrm{erfc}\left(\left(2i+1\right)\sqrt{\frac{3\;\cdot \;\rho_{n}\;\cdot \;\gamma_{n}}{2(M^{2}-1)}}\right)\right\}\right]\right]\\ \; & =\frac{1}{M\log_{2}M}\;\sum_{k=1}^{\log_{2}M}\left[\sum_{i=0}^{(1-2^{-k})M-1}\;\;\left\{\;\Phi \left(i,k,m\right)\;\cdot \;E_{\gamma_{n}}\;\left[\mathrm{erfc}\left(\left(2i+1\right)\sqrt{\frac{3\;\cdot \;\rho_{n}\;\cdot \;\gamma_{n}}{2(M^{2}-1)}}\right)\right]\right\}\right], \end{array}$$

where $\rho_{n}\;\left(=P_{n}/N_{0}\right)$ denotes the transmit SNR of the *n*th STA and

$$\Phi \left(i,k,M\right)={\left(-1\right)}^{\left\lfloor \;\frac{i\cdot 2^{k-1}}{M}\;\right\rfloor }\left(2^{k-1}-\left\lfloor \frac{i\cdot 2^{k-1}}{M}+\frac{1}{2}\right\rfloor \right),$$

where ⌊·⌋ denotes the floor function that gives the greatest integer number less than or equal to the input real number. Furthermore, we define a random variable for the effective channel gain of the STA as $X_{n}:=\gamma_{n}$. The probability density function of $X_{n}$ is given by $f_{X_{n}}\left(x_{n}\right)=\left(x_{n}/\beta_{n}^{2}\right)\exp\left(-x_{n}/\beta_{n}\right)$ from the definition of $\gamma_{n}$; hence the expectation term in (6) can be derived as follows:

(7) $$\begin{array}{cc} E_{\gamma_{n}} & \left[\mathrm{erfc}\left(\left(2i+1\right)\sqrt{\frac{3\cdot \rho_{n}\cdot \gamma_{n}}{2(M^{2}-1)}}\right)\right]=\int_{0}^{\infty }\mathrm{erfc}\left(\left(2i+1\right)\sqrt{\frac{3\cdot \rho_{n}\cdot x_{n}}{2(M^{2}-1)}}\right)f_{X_{n}}\left(x_{n}\right)dx_{n}\\ \; & =\int_{0}^{\infty }\mathrm{erfc}\left(\left(2i+1\right)\sqrt{\frac{3\cdot \rho_{n}\cdot x_{n}}{2(M^{2}-1)}}\right)\frac{x_{n}}{\beta_{n}^{2}}\exp\left(-\frac{x_{n}}{\beta_{n}}\right)dx_{n}\\ \; & =1-\frac{\sqrt{\frac{3{\left(2i+1\right)}^{2}\cdot \beta_{n}\cdot \rho_{n}}{2(M^{2}-1)}}}{\sqrt{\frac{3{\left(2i+1\right)}^{2}\cdot \beta_{n}\cdot \rho_{n}}{2(M^{2}-1)}+1}}\left[1+\frac{1/2}{\frac{3{\left(2i+1\right)}^{2}\cdot \beta_{n}\cdot \rho_{n}}{2(M^{2}-1)}+1}\right]. \end{array}$$

By substituting (7) into (6), the analytical BER performance closed-form for the *n*th STA in the proposed low-complexity uplink STLC-NOMA network is obtained. It is worth noting that each STA achieves the same BER performance as using orthogonal resources.

Moreover, the expectation term can be approximated by using Taylor series expansion under the high SNR regime as follows:

(8) $$E_{\gamma_{n}}\left[\mathrm{erfc}\left(\left(2i+1\right)\sqrt{\frac{3\cdot \rho_{n}\cdot \gamma_{n}}{2(M^{2}-1)}}\right)\right]\approx \frac{3}{8{\left(\frac{3{\left(2i+1\right)}^{2}\cdot \beta_{n}\cdot \rho_{n}}{2(M^{2}-1)}\right)}^{2}}=\frac{1}{6}{\left(\frac{M^{2}-1}{{\left(2i+1\right)}^{2}\cdot \beta_{n}}\right)}^{2}\rho_{n}^{-2}.$$

By substituting (8) into (6), the asymptotic BER performance of the *n*th STA is obtained, which represents the BER behavior in the high SNR regime.

Finally, from the asymptotic BER expression, we can derive the spatial diversity order of each STA in the proposed STLC-NOMA system as follows:

(9) $$\eta_{n}≜-\lim_{\rho_{n}\to \infty }\frac{\log P_{b,n}}{\log\rho_{n}}=2.$$

We can clearly state that the proposed uplink STLC-NOMA system achieves the optimal spatial diversity order of two for both STAs.

## 4. Blind Energy Estimation (BEE)

**Figure 1:**
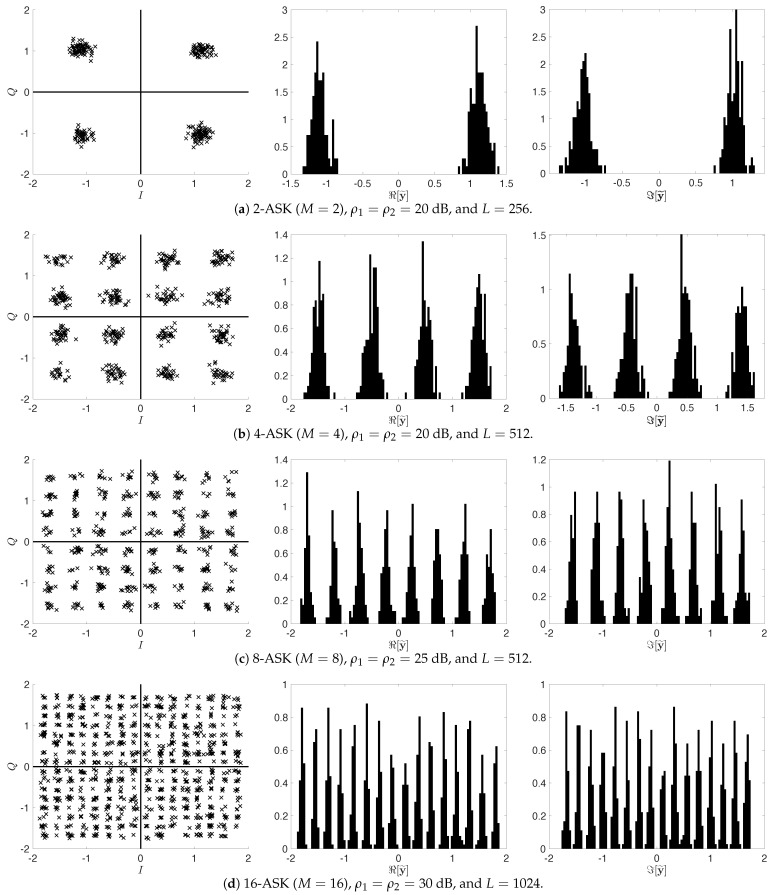
The constellation diagrams of $\tilde{y}$ (first column), in-phase axis energy histograms $ℜ[\tilde{y}]$ (second column), and quadrature axis energy histograms $ℑ[\tilde{y}]$ (third column), for M=2,4,8,16, respectively, where $P_{1}=P_{2}=1$, $\gamma_{1}\approx 1.1032$, and $\gamma_{2}\approx 1.0303$. For each case, the system SNR $\rho_{n}$ and frame length *L* were set appropriately to clearly present the characteristics.

We can observe from (4) that the STLC decoded signal at the AP ${\tilde{y}}_{l}$ conforms to an i.i.d. mixture of Gaussian distributions

$${\tilde{y}}_{l}∼\frac{1}{{\left|X\right|}^{2}}\sum_{x_{n}^{\left(l\right)}\in X}\mathrm{CN}\left(\sqrt{P_{1}\gamma_{1}}x_{1}^{\left(l\right)}+j\sqrt{P_{2}\gamma_{2}}x_{2}^{\left(l\right)},2N_{0}\right),$$

because this is affected by AWGN as illustrated in the first column in Figure 1. Furthermore, since the signals of both STAs are aligned on the in-phase and quadrature axes, respectively, they can be divided into two components as follows:

(10) $$\begin{array}{cc} ℜ[{\tilde{y}}_{l}]\; & ∼\;\frac{1}{\left|X\right|}\;\sum_{x_{1}^{\left(l\right)}\in X}\;N\left(\sqrt{P_{1}\gamma_{1}}x_{1}^{\left(l\right)},N_{0}\right),\;\;\mathrm{for}\;\mathrm{the}\;\mathrm{first}\;\mathrm{STA},\\ ℑ[{\tilde{y}}_{l}]\; & ∼\;\frac{1}{\left|X\right|}\;\sum_{x_{2}^{\left(l\right)}\in X}\;N\left(\sqrt{P_{2}\gamma_{2}}x_{2}^{\left(l\right)},N_{0}\right),\;\;\mathrm{for}\;\mathrm{the}\;\mathrm{second}\;\mathrm{STA}, \end{array}$$

as shown in the second and third columns in Figure 1. It is worth noting that each of (10) still follows a mixture of Gaussians and can be modeled as a one-dimensional (1D) Gaussian mixture model (GMM).

We now design an expectation-maximization (EM)-based blind energy estimation (BEE) scheme for blindly estimating the transmit power and effective channel gain, $P_{n}\gamma_{n}$, of each STA at the AP. Briefly, we exploit the EM algorithm [24], also known as soft *K*-means clustering, which infers the parameters of the GMM given the number of STAs and modulation type of each STA [25,26]. Recall that although the constellation consisting of the superimposed *M*-ASK symbols from the two STAs configures $M^{2}$ clusters as shown in the first column in Figure 1, the signals of each STA are aligned on each axis. Hence, we consider 1D GMMs with *M* parameters for the real and imaginary axes in (10).

Algorithm 1 represents the pseudo-code of the proposed BEE scheme for the two-user uplink STLC-NOMA system employing *M*-ASK modulation, where $N(z|\mu ,\sigma^{2})$ denotes a Gaussian probability density function with mean μ and variance $\sigma^{2}$ expressed as

(11) $$N\left(z|\mu ,\sigma^{2}\right)=\frac{1}{\sqrt{2\pi \sigma^{2}}}\exp\left(-\frac{{\left(z-\mu \right)}^{2}}{2\sigma^{2}}\right).$$

The AP executes this algorithm by taking the STLC decoding signals $\tilde{y}$ in (4) as observed data to infer the parameters of a GMM compromising *M* means, variances, and weights for each axis. It can be stated that the objective of Algorithm 1 is to maximize the likelihood function with respect to the GMM parameters for all observations,

(12) $$\mathrm{Pr}\left(z|\mu_{1},\mu_{2},\cdots ,\mu_{M},\sigma^{2}\right)=\prod_{l=1}^{L}\sum_{k=1}^{M}\pi_{k}N\left(z_{l}|\mu_{k},\sigma^{2}\right),$$

where $z\;(={\left[z_{1},\cdots ,z_{L}\right]}^{T}\in R^{L\times 1})$ represents the observations, $\mu_{k}$ and $\pi_{k}$ denote the mean and weight of the *k*th Gaussian distribution, respectively, and $\sum_{k=1}^{M}\pi_{k}=1$. Although the canonical EM algorithm for a GMM infers the mean $\mu_{k}$, variance $\sigma_{k}^{2}$, and weight $\pi_{k}$ of each Gaussian distribution [24], we exploit a shared common variance $\sigma^{2}$ for all Gaussian distributions in a GMM. This is because all symbols in a frame experience AWGN with the same variance, so the clusters formed at the AP may have almost the same density. More specifically, Algorithm 1 is as follows.
**Algorithm 1** Blind energy estimation (BEE) algorithm.  1:$\mathrm{Input}:\tilde{y}$.  2:$\mathrm{Output}:\hat{P_{1}\gamma_{1}},\;\hat{P_{2}\gamma_{2}}$.  3:**for all**n(∈{1,2})**do**  4:    $z=\left\{\begin{array}{ll} ℜ[\tilde{y}], & \mathrm{if}\;n=1,\\ ℑ[\tilde{y}], & \mathrm{if}\;n=2. \end{array}\right.$  5:    Initialize the means $\mu_{1},\cdots ,\mu_{M}$ and variance $\sigma^{2}$ by dividing z into *M* clusters,    $\pi_{k}=1/M$, ∀k∈{1,⋯,M}, and $F_{\mathrm{old}}=0$  6:    Calculate log-likelihood $F_{\mathrm{new}}=\sum_{l=1}^{L}\ln\left[\sum_{k=1}^{M}\pi_{k}N\left(z_{l}|\mu_{k},\sigma^{2}\right)\right]$  7:    **while** $|F_{\mathrm{new}}-F_{\mathrm{old}}|\geq \epsilon$ **do**           ▹ ***Expectation (E-Step)***  8:         Update responsibilities $\left.r_{k}^{\left(l\right)}=\pi_{k}N\left(z_{l}|\mu_{k},\sigma^{2}\right)/\sum_{k^{\prime }=1}^{M}\pi_{k^{\prime }}N\left(z_{l}|\mu_{k^{\prime }},\sigma^{2}\right)\right.$, ∀k,l         ▹ ***Maximization (M-Step)***  9:         Update means $\left.\mu_{k}=\sum_{l=1}^{L}r_{k}^{\left(l\right)}z_{l}/\sum_{l=1}^{L}r_{k}^{\left(l\right)}\right.$, ∀k 10:        Update variance $\left.\sigma^{2}=\sum_{k=1}^{M}\left(\sum_{l=1}^{L}r_{k}^{\left(l\right)}{\left(z_{l}-\mu_{k}\right)}^{2}/\sum_{l=1}^{L}r_{k}^{\left(l\right)}\right)/M\right.$ 11:        Update weights $\left.\pi_{k}=\sum_{l=1}^{L}r_{k}^{\left(l\right)}/\sum_{k=1}^{M}\sum_{l=1}^{L}r_{k}^{\left(l\right)}\left(=\sum_{l=1}^{L}r_{k}^{\left(l\right)}/L\right)\right.$, ∀k 12:        Update log-likelihood $F_{\mathrm{old}}\leftarrow F_{\mathrm{new}}$ 13:        Calculate log-likelihood $F_{\mathrm{new}}=\sum_{l=1}^{L}\ln\left[\sum_{k=1}^{M}\pi_{k}N\left(z_{l}|\mu_{k},\sigma^{2}\right)\right]$ 14:    **end while** 15:    Estimate $\sqrt{\hat{P_{n}\gamma_{n}}}=\frac{\sqrt{M\sum_{m=1}^{M}{\left(2m-1-M\right)}^{2}}}{\sum_{m=1}^{M}\left|2m-1-M\right|}\sum_{k=1}^{M}\pi_{k}\left|\mu_{k}\right|$ 16:**end for**

### 4.1. Initialization (Lines 5 to 6)

The AP divides observations z into *M* sets in descending order, each with the same number of elements, and calculates the means and variance by taking each set as a cluster. As aforementioned, we consider the common variance for each cluster, which is obtained by taking an average over the variances for all clusters. The weights are initialed by $\pi_{k}=1/M$ for all clusters since all constellation points of the STA tend to be uniformly drawn due to purely random bit generations. Then, the log-likelihood function is calculated from the initial GMM parameters and observations.

### 4.2. EM-Step (Lines 7 to 14)

The EM is an iterative two-step algorithm in which the expectation phase (E-step) evaluates the responsibilities using the current parameters, and the maximization phase (M-step) updates the parameters using the current responsibilities. Specifically, in *E-Step*, the responsibilities $r_{k}^{\left(l\right)}$ are evaluated for all k(∈{1,2,⋯,M}) and l(∈{1,2,⋯,L}) (line 8). As the name implies, each value of this indicates the responsibility of cluster *k* for observation *l*. Then, in *M-Step*, each cluster’s mean $\mu_{k}$, variance $\sigma^{2}$, and weight $\pi_{k}$ are adjusted to match the observations it is responsible for (lines 9 to 13). Note that we consider a common variance for all clusters and draw it by averaging *M* variances. Then, the AP evaluates the log-likelihood and compares it to the previous log-likelihood to check convergence. If it does not converge, repeat from E-step.

### 4.3. Energy Estimation (Line 15)

Finally, the received energy $P_{n}\gamma_{n}$ for each STA is estimated from the inferred means and weights, where the equation is derived from the following procedure. Without loss of generality, assuming *μ*_1_ < *μ*_2_ < ⋯ < *μ*_*M*_, the inferred means can correspond to the normalized *M*-ASK constellation points as follows:

(13) $$\mu_{k}=\frac{2k-1-M}{\sqrt{\sum_{m=1}^{M}{\left(2m-1-M\right)}^{2}/M}}\sqrt{P_{n}\gamma_{n}},$$

The AP is interested in estimating the received energy of the STA $\sqrt{P_{n}\gamma_{n}}\;\left(\geq 0\right)$ from the inferred constellation points, which can be obtained through the following equation:

(14) $$\frac{1}{M}\sum_{k=1}^{M}\left|\mu_{k}\right|=\frac{1}{M}\sum_{k=1}^{M}\frac{\left|2k-1-M\right|}{\sqrt{\sum_{m=1}^{M}{\left(2m-1-M\right)}^{2}/M}}\sqrt{P_{n}\gamma_{n}}.$$

Here, 1/M on the left-hand side represents the weight assuming all clusters have the same weights, i.e., $\mu_{k}=1/M,\;\forall k$. However, in practice, each point on the constellation may not be expressed with exactly the same probability depending on the generated bit sequence. Hence, the AP can estimate the received energy of the STA from the inferred means and weights as

(15) $$\sum_{k=1}^{M}\pi_{k}\left|\mu_{k}\right|=\frac{\sum_{k=1}^{M}\left|2k-1-M\right|}{\sqrt{M\sum_{m=1}^{M}{\left(2m-1-M\right)}^{2}}}\sqrt{P_{n}\gamma_{n}}.$$

Finally,

(16) $$\sqrt{P_{n}\gamma_{n}}=\frac{\sqrt{M\sum_{m=1}^{M}{\left(2m-1-M\right)}^{2}}}{\sum_{m=1}^{M}\left|2m-1-M\right|}\sum_{k=1}^{M}\pi_{k}\left|\mu_{k}\right|.$$

## 5. Simulation Results

**Figure 2:**
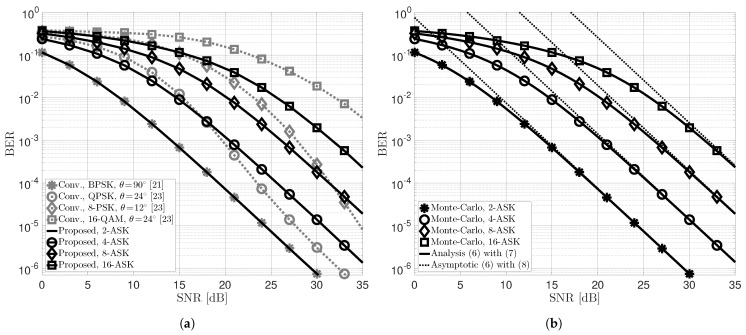
Average BER performance of two STAs in the proposed low-complexity uplink STLC-NOMA and conventional STLC-NOMA systems. (**a**) Numerical BER performance of the proposed STLC-NOMA (Proposed) and conventional STLC-NOMA (Conv.) [23]. (**b**) Analytic and asymptotic BER performance of the proposed STLC-NOMA.

The BER performance of the proposed novel low-complexity two-user uplink STLC-NOMA system and its mathematical expressions in Section 3 were verified through extensive computer simulations, as shown in Figure 2. Here, we assumed that each STA’s transmit power and partial CSI, denoted by $P_{n}\gamma_{n}$ (received energy), are perfectly estimated at the AP, and set the transmit powers and large-scale fading components of the two STAs to $P_{1}=P_{2}=P$ and $\beta_{1}=\beta_{2}=1$, respectively. Figure 2a compares the BER performance of the proposed novel uplink STLC-NOMA (Proposed) with the conventional uplink STLC-NOMA (Conv.) [23], considering the same modulation order *M*. Specifically, for a fair comparison, we compared the proposed STLC-NOMA systems employing 2, 4, 8, and 16-ASK with the conventional STLC-NOMA systems using binary-PSK (BPSK), quadrature-PSK (QPSK), 8-PSK, and 16-QAM, respectively. Because BPSK with an optimal constellation rotation angle of 90° in the conventional STLC-NOMA [21] and 2-ASK in the proposed STLC-NOMA have the same constellation diagram, both have the same BER performance. Somewhat interestingly, the proposed two-user uplink STLC-NOMA system has improved BER performance in the low SNR regime, and this trend becomes more remarkable as the modulation order increases. For modulation orders of M=4 and 8, the BER performance intersects at SNRs of approximately 18 and 31.5 dB, respectively, and for M=16, the proposed STLC-NOMA outperforms the conventional STLC-NOMA in the entire SNR regime. For M=4, the conventional STLC-NOMA is about 3 dB better than the proposed STLC-NOMA in terms of the SNR to satisfy the same BER in the high SNR regime. As discussed in Section 2, however, it is also worth noting that the proposed STLC-NOMA has significantly lower computational complexity at the AP than the conventional STLC-NOMA because it does not employ a joint detector. Figure 2b validates the mathematical BER performance analysis results (6)–(8) in Section 3. It can be observed that the analytical BER expression is precisely the same as the Monte-Carlo simulation results for arbitrary modulation order. Moreover, the asymptotic BER expression, exploited to reveal that the proposed uplink STLC-NOMA system achieves optimal spatial diversity order for both STAs, is also well-matched with the simulation results.

**Figure 3:**
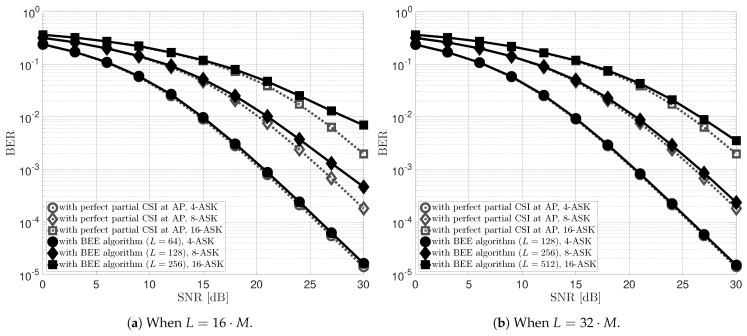
Average BER performance of two STAs in the proposed low-complexity uplink STLC-NOMA system employing the BEE algorithm at the AP with respect to the frame length.

**Figure 4:**
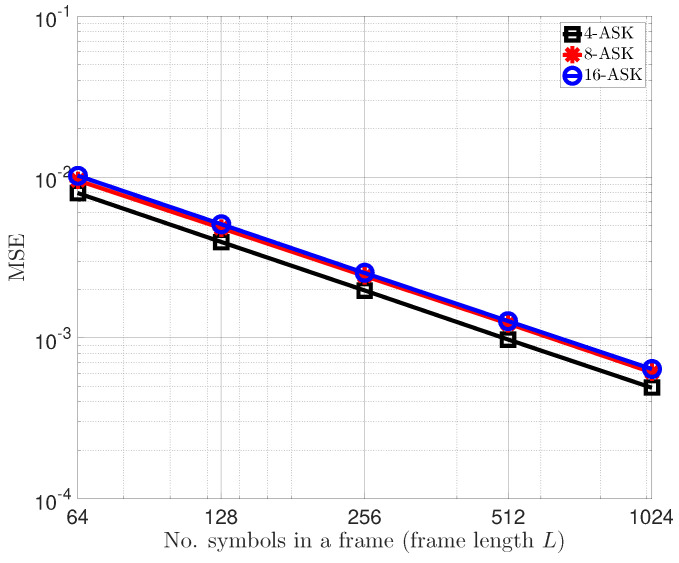
Average MSE of the proposed BEE algorithm with respect to the frame length, when the transmit SNRs of both STAs are $\rho_{1}=\rho_{2}=30$ dB.

Figure 3 shows the BER performance of the proposed uplink STLC-NOMA system with the BEE scheme designed in Section 4. Here, 2-ASK is omitted because even the channel gain is not required in this case like the PSK constellation diagram. Specifically, as shown in the first column in Figure 1a, the AP can detect the signals transmitted from STAs by distinguishing only the quadrant. The transmit powers and large-scale fading components of both STAs are still set to $P_{1}=P_{2}=P$ and $\beta_{1}=\beta_{2}=1$, respectively. For the BEE algorithm, the maximum number of iterations of the EM loop was set to 100, and $\epsilon =10^{-8}$. Moreover, we set the frame length of each STA to L=16·M in Figure 3a and L=32·M in Figure 3b, according to the modulation order *M*. We can observe from Figure 3 that since the accuracy of the clustering method depends on the number of available data points (the number of symbols in a frame), as shown in Figure 4, the longer the frame length, the better the BER performance. For example, with an SNR of 30 dB and modulation orders of 8 and 16, the BER performance is improved by almost 50% when L=32·M compared to when L=16·M. On the other hand, the proposed BEE algorithm has a trade-off between accuracy and frame length. Specifically, the BEE algorithm has O(L·M) computational complexity depending on the frame length *L* and modulation order *M*; hence the complexity linearly increases as the frame length increases. Figure 4 depicts the average mean squared error (MSE) of the estimated energies for both STAs, defined as $\mathrm{MSE}:=\sum_{n=1}^{2}{\left(\sqrt{\gamma_{n}}-\sqrt{{\hat{\gamma }}_{n}}\right)}^{2}/2$, when the transmit SNRs of the two STAs are 30 dB, i.e., $\rho_{1}=\rho_{2}=30$ dB. We can observe that the MSE of the BEE algorithm has linearly improved with respect to the frame length in the log-log domain. As earlier mentioned, the more symbols within a frame, the more observations available at the AP, resulting in the MSE improvement. Figure 3 and Figure 4 also show that the designed BEE scheme works well enough for short-length frames.

**Figure 5:**
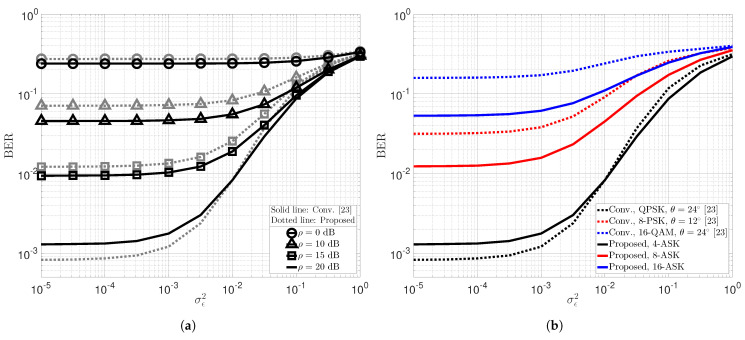
Average BER performance of two STAs in the proposed low-complexity uplink STLC-NOMA and conventional STLC-NOMA systems with channel estimation error. (**a**) The proposed STLC-NOMA with 4-ASK and conventional STLC-NOMA with QPSK for varying SNRs. (**b**) The proposed STLC-NOMA with *M*-ASK and conventional STLC-NOMA with *M*-PSK/QAM for an SNR of 20 dB.

Meanwhile, CSI estimation errors may exist in practical wireless networks, even in channel estimation through pilot signals at the STA. Such a CSI estimation error can be statistically modeled as ${\tilde{h}}_{n,m}≜h_{n,m}+\epsilon_{n,m}$, where ${\tilde{h}}_{n,m}$ and $h_{n,m}$ represent the actual CSI and the estimated CSI, respectively, and $\epsilon_{n,m}$ indicates the estimation error [23]. In general, the resultant estimation error is assumed to follow an i.i.d. $\mathrm{CN}(0,\sigma_{\epsilon }^{2}),\;\forall n,m$, where variance $\sigma_{\epsilon }^{2}$ represents the MSE of the estimation such that $E[|h_{n,m}-{\tilde{h}}_{n,m}\left|\right]=\sigma_{\epsilon }^{2}$. Figure 5 shows the average BER performance of the two STAs in the uplink STLC-NOMA systems with respect to the MSE of the channel estimation, where $\rho_{1}=\rho_{2}=\rho$, $\beta_{1}=\beta_{2}=1$, and L=32·M. In particular, Figure 5a shows the results for varying SNRs when the modulation order is 4, and Figure 5b presents the results for varying modulation orders when the SNR is 20 dB. The proposed STLC-NOMA employs a blind receiver, whereas the conventional STLC-NOMA assumes that the AP has perfect partial CSI because there is no proposed blind receiver. As expected, we can observe that the BER performance of the STLC-NOMA systems gradually deteriorates as the MSE of estimation increases. It is worth noting that the BER performance is crossed when ρ=20 dB and $\sigma_{\epsilon }^{2}\approx 10^{-2}$ in Figure 5a, even though the perfect partial CSI is assumed at the AP for the conventional STLC-NOMA. Hence, we can state that the proposed low-complexity STLC-NOMA system is more tolerant of channel estimation errors than the conventional STLC-NOMA system.

## 6. Conclusions

We have proposed a novel low-complexity STLC-NOMA system for two-user uplink IoT networks. In summary, both STAs employ the *M*-ASK modulator and align their modulated symbols to each axis in the I-Q plane, respectively, before STLC encoding. Thanks to the characteristics of the STLC that remove the phase distortion caused by wireless channels, the signals of each STA are still aligned on their axis at the AP. Hence, the AP can detect the transmitted signals through the ML detector without joint detection. Simulation results have shown that the proposed novel uplink STLC-NOMA system is more efficient than the conventional uplink STLC-NOMA in terms of the computational complexity of the detector and BER performance under the low SNR regime. Furthermore, we have mathematically analyzed the exact BER performance of each STA in the proposed uplink STLC-NOMA system, and verified that the optimal spatial diversity order could be achieved. We also validated that these mathematical BER expressions are exactly the same as the Monte-Carlo simulation results. Finally, we have designed an EM-based blind energy estimation (BEE) scheme to blindly estimate the transmit power and effective channel gain of each STA at the AP and observed that it works well even for short packet transmission.

## Data Availability

Not applicable.

## Abbreviations

The following abbreviations are used in this manuscript:

AP	Access point
ASK	Amplitude-shift keying
AWGN	Additive white Gaussian noise
BEE	Blind energy estimation
BER	Bit-error-rate
CSI	Channel state information
CSIT	Channel state information is available only at transmitter
EM	Expectation-maximization
GMM	Gaussian mixture model
I-Q	In-phase and quadrature
i.i.d.	independent and identically distributed
MIMO	Multiple-input multiple-output
ML	Maximum-likelihood
NOMA	Non-orthogonal multiple access
PAM	Pulse amplitude modulation
PSK	Phase-shift keying
SNR	Signal-to-noise ratio
STA	Station (IoT device)
STLC	Space-time line code
STLC-NOMA	Space-time line coded non-orthogonal multiple access
